# Validation and Evaluation of the Chinese Version of the Healthy Aging Questionnaire in Hong Kong

**DOI:** 10.1155/jare/3632426

**Published:** 2026-04-08

**Authors:** Jed Montayre, Wenjing Ning, Ka Man Carman Leung, Chun Hei Glen Cheng, Fen Liu, Juliet Chigozie Donatus Ezulike, Kay Kuo

**Affiliations:** ^1^ School of Nursing, The Hong Kong Polytechnic University, Hong Kong SAR, China, polyu.edu.hk

**Keywords:** healthy aging, psychometrics, reliability, validity

## Abstract

**Background:**

Healthy aging is a key public health priority in Hong Kong. The Healthy Aging Questionnaire, originally developed and validated in Singapore for older adults, is a concise tool for assessing multiple dimensions of healthy aging.

**Objectives:**

This study examines the psychometric performance of the Chinese version of the HAQ in the Hong Kong adult population, assessing validity, reliability, and factor structure.

**Research Designs and Methods:**

A cross‐sectional study was conducted from October to December 2024 using convenience sampling to recruit participants aged 18 and above from various communities in Hong Kong through online and offline methods. Exploratory factor analysis and confirmatory factor analysis were performed to determine the factor structure. Cronbach’s alpha, split‐half reliability, and intraclass correlation coefficient were used to assess reliability.

**Results:**

The Chinese version of the HAQ demonstrated face validity and content validity. An exploratory factor analysis confirmed a three‐factor structure, comprising physical/mental and sense of purpose, engagement in functional activities, and engagement in social and physical activities, with a cumulative variance contribution of 58.178%. The instrument showed Cronbach’s alpha was 0.827, split‐half reliability was 0.697, and an intraclass correlation coefficient was 0.630. Confirmatory factor analysis validated the three‐factor structure.

**Discussion:**

The Chinese version of the HAQ is a valid and reliable tool for assessing healthy aging in the Hong Kong adult population.

**Implications:**

The robust psychometric properties support its use in research and practice to evaluate and promote healthy aging. Future studies should validate the HAQ in longitudinal research.

## 1. Introduction

### 1.1. Background

The World Health Organization (WHO) defines healthy aging as the continuous process of fostering and preserving the functional capacity that supports well‐being in later life [1]. Functional ability refers to individuals’ capacities to meet their basic needs, learn and grow, make decisions, maintain mobility, build relationships, and contribute to society [1]. As the global population ages rapidly, it is essential to understand that the determinants of healthy aging have become a critical public health priority [2]. It holds implications for improving the quality of life for older adults, reducing healthcare costs, and promoting active community engagement [3].

In Hong Kong, like many other places worldwide, the population is aging at an unprecedented rate. In 2015, 21.7% of Hong Kong’s population was aged 60 or above, making it the second‐highest in Asia after Japan [4]. Projections for 2012–2041 indicate that this percentage will rise to 36.3% by 2041 [4]. This demographic shift brings about significant challenges in terms of healthcare, social services, and public policy. However, it is equally important to focus on promoting healthy aging among adults in general, not just for those over 65. As the adult population continues to age, addressing the health and well‐being needs of individuals across the entire adulthood life course (from young adulthood to older adulthood) has become a growing priority [5].

Along with the significant effort to promote healthy aging, the goal is to accurately measure indicators of healthy aging. The Healthy Aging Questionnaire (HAQ) was developed in Singapore and offered a concise and comprehensive instrument for assessing multiple aspects of healthy aging, including physical function, cognitive health, emotional well‐being, and social engagement [6]. The original HAQ was designed to assess healthy aging in older adults aged 65 and above. In the current study, healthy aging is a lifelong process shaped by various factors across different stages of adulthood. Health behaviors and lifestyle choices in early and mid‐life play a critical role in determining aging outcomes [7]. Studies indicate that maintaining a physically active lifestyle and managing stress during early adulthood are associated with improved cognitive function [8] and lower risk of chronic diseases [9]. Additionally, mental well‐being in midlife is associated with a reduced risk of dementia in later adulthood [10]. Although Cantonese is spoken in both Hong Kong and Singapore, Cantonese is the dominant language in Hong Kong, whereas in Singapore, it is less commonly used, with Mandarin and English being more prevalent, resulting in differences in vocabulary, cultural expressions, and perceptions of aging. These linguistic and cultural differences necessitate the validation of the HAQ in Hong Kong to ensure its accuracy and relevance for the local population. Given that the participants in this study are adults aged 18 and older, it is important to determine the performance of HAQ in the broader adult population, considering that healthy aging indicators may vary across different stages of adulthood. For instance, younger adults might place more emphasis on work–life balance or mental health, while older adults may focus more on mobility and social support. Therefore, to ensure the instrument’s relevance and accuracy for the Hong Kong adult population, it is crucial to translate and validate the HAQ in Chinese, the primary language spoken by the majority of residents in Hong Kong.

By translating and validating the HAQ in Chinese, researchers and healthcare professionals in Hong Kong will be equipped with a culturally appropriate tool to assess the health status and aging process of adults. This will enable the identification of areas where intervention is needed to improve the quality of life for adults of all ages, support their physical and mental health, and enhance their social participation. Furthermore, a validated Chinese version of the HAQ can contribute to broader research on healthy aging in Hong Kong, informing public health policies, community programs, and interventions aimed at promoting healthy aging in this rapidly aging population.

### 1.2. Aim

This study aims to examine the psychometric performance of the translated Chinese version of the HAQ in the Hong Kong adult population.

## 2. Methods

### 2.1. Study Design

The research report followed the Strengthening the Reporting of Observational Studies in Epidemiology (STROBE) guidelines [11] and guidelines for presenting the results of instrument and scale development and testing studies [12].

To translate and validate the Chinese version of the HAQ, we conducted a cross‐sectional study in Hong Kong from October to December 2024. This study used convenience sampling to recruit participants from different communities in the city through online and offline recruitment methods. Participants were included if they were: (a) aged 18 or above and (b) currently residing in Hong Kong. Individuals were excluded if they: (a) lacked English or Chinese language proficiency and (b) had conditions that potentially impeded their mental capacity to understand the study’s objectives and provide consent to participate.

### 2.2. HAQ

The15‐item HAQ was originally developed in Singapore as a concise and practical measure derived from the 280‐item Singapore Successful Aging Questionnaire (SSOSA) [6].

### 2.3. Translation Procedure

The translation of the HAQ into Chinese followed Brislin’s translation method [13]. The process consisted of the following phases: (a) Initial Consent and Preparation: The research team first obtained the original developers of the HAQ to translate the instrument into Chinese for this study. (b) Forward Translation: Two bilingual translators, whose mother tongue is Chinese, independently translated the HAQ from English into traditional Chinese. One of the translators had a nursing background, and the other had a nonmedical background. Any discrepancies were further discussed with the research team to produce a consensus‐based initial draft. (c) Back‐Translation: The translated draft was then back‐translated into English by two independent translators who were proficient in both languages but unfamiliar with the original HAQ. This step ensured that the translated version retained the questionnaire’s original meaning and intent. (d) Comparison and Reconciliation: The research team compared the back‐translated version with the original HAQ to identify and resolve any meaningful inconsistencies or deviations. Adjustments were made to the translated draft to ensure conceptual equivalence. (e) Cultural Adaptation: To ensure cultural relevance and appropriateness, the translated draft was reviewed by a panel of experts, including healthcare professionals, researchers, and educators with extensive experience in aging‐related studies. These experts evaluated the translated items for conceptual equivalence, content relevance, and clarity. Any ambiguous or culturally inappropriate terms were revised based on their feedback. (f) Pilot Testing: The culturally adapted version of the HAQ was pilot‐tested with 3 individuals aged from 45 to 60. Feedback from this pilot test was used to identify and resolve any remaining misunderstandings or ambiguities in the translated items. (g) Finalization: After incorporating feedback from the pilot test, the Chinese version of the translated HAQ was produced and ready for use in this study. (h) Rationale for Participant Selection in Language Validation: While the HAQ was originally designed for older adults, the language validation (including expert panel review and pilot cognitive interviews) targeted a wider adult population (18–65 years). This decision aligns with the study’s lifelong perspective on healthy aging, which recognizes that the determinants of healthy aging span the adult life course and the need to establish the instrument’s relevance to the broader target population of this study (adults aged 18 years and above). Additionally, this approach was designed to capture potential generational differences in language use and the cultural interpretation of concepts within the Hong Kong Cantonese context. Importantly, it enabled the identification of underlying language and comprehension issues before testing potentially frail older adults in the subsequent psychometric validation phase. The subsequent psychometric validation phase (exploratory factor analysis/confirmatory factor analysis [EFA/CFA]) utilized the full baseline cohort data (*N* = 2024), which included a significant proportion of older adults, to rigorously evaluate the instrument’s properties in the target older population.

This comprehensive translation process ensured that the HAQ maintained its original meaning, conceptual integrity, and cultural relevance while adapting to a new linguistic and cultural context.

### 2.4. Data Collection

Participants completed the questionnaire using either online forms (Google Forms) or paper copies. All the participants answered the HAQ and basic demographic information. A total of 2120 responses were collected through paper surveys and online forms (Google Forms). Of these, 70 responses were obtained through paper surveys, and 2050 through online forms. Among the online responses, there were four invalid responses (resulting from internal testing), 90 duplicates, and one response from a participant who withdrew from the study. Additionally, one missing data point (less than 0.05%) was identified and removed from the paper survey responses. After these exclusions, 2024 valid responses were retained for analysis. Multiple recruitment strategies were employed, including personal networks and referrals from family and friends, distribution of promotional posters, targeted visits to care centers, classroom invitations, email promotions, postal contact, and online advertising campaigns. All surveys were distributed in Traditional Chinese to ensure accessibility and clarity for the target population. This comprehensive approach facilitated broad participation and ensured the reliability and validity of the collected data. The detailed data collection procedure is summarized in the flow diagram provided in Supporting Figure S1.

### 2.5. Data Analysis

(EFA) and descriptive analysis were conducted in IBM SPSS Statistics v.27.0 and CFA in IBM SPSS Amos v.28.0 (SPSS, Chicago, IL, USA). The participants’ characteristics were interpreted using descriptive analyses, which included mean, standard deviation, median, frequencies, and value ranges.

#### 2.5.1. Content Validity

The seven experts involved in the cultural adaptation phase were invited to evaluate the adapted content using a 4‐point Likert scale (ranging from 1 = not relevant to 4 = very relevant). Content validity was considered acceptable if the item‐level content validity index (I‐CVI) was ≥ 0.80 and the scale‐level content validity index (S‐CVI) was ≥ 0.90 [14], provided that at least six experts participated in the evaluation.

#### 2.5.2. Item Analysis

Item analysis was conducted to evaluate and refine the items of the HAQ, ensuring their quality and relevance [15]. The analysis employed several evaluation techniques, including the correlation coefficient method, Cronbach’s *α*, and factor loading (through factor analysis). Items were considered for elimination based on the following criteria: (a) a corrected item‐total correlation coefficient value of less than 0.40 [16]; (b) a significant increase in Cronbach’s *α* when the item was removed [17]; and (c) a corrected item‐total correlation, with values greater than or equal to 0.30 considered acceptable [18]. However, there was a tolerance to an item with a corrected item‐total correlation above 0.2 [19]. Items meeting one of these criteria were flagged for potential removal. The research team made final decisions on elimination, incorporating professional judgment and assessment to ensure the integrity and validity of the HAQ.

#### 2.5.3. Factor Analysis

EFA and CFA were conducted on separate samples to prevent overfitting and ensure the factor structure’s reliability, validity, and generalizability across diverse datasets [20]. The dataset was randomly divided into two parts (sample A and sample B) to perform EFA and CFA separately. The randomization was conducted using Microsoft Excel, where each case was assigned a randomly generated number, and the dataset was then split into two groups based on these values.

Sample A was used to conduct the EFA to explore the underlying factor structure. To evaluate the values of the data for EFA, the Kaiser–Meyer–Olkin (KMO) value of ≥ 0.8 indicated adequate sampling [21], and Bartlett’s test of sphericity with a *p*‐value < 0.05 indicated that the variables in the dataset did not form an identity matrix [22], ensuring that the factor analysis results were satisfactory. EFA was conducted using the principal axis factoring (PAF) procedure [23]. PAF with Varimax rotation was performed to determine the number of factors within the scale, using a factor loading of ≥ 0.4 to indicate significance [16]. Additionally, there were no cross‐loading item loads at 0.32 or higher on two or more factors [24]. An initial factor structure analysis, based on eigenvalues more significant than 1, was performed to optimize the number of factors to be extracted through a scree plot [25]. Given the theoretical expectation that domains of healthy aging are correlated, Promax (oblique) rotation was applied [26]. Items with factor loadings ≥ 0.50 were assigned to the identified factors, and interfactor correlations were examined.

Sample B was used to conduct the CFA to explore the underlying factor structure. The CFA used structural equation modeling to test a hypothesized model for the goodness of fit to the actual data. Fit indices selected in this study were the incremental fit index (IFI), the Tucker–Lewis index (TLI), and the comparative fit index (CFI). A value ≥ 0.9 indicated a reasonable fit [27], and a value of ≥ 0.95 was considered an excellent fit [28]. In addition, the value of the root‐mean‐square error of approximation (RMSEA) < 0.08 indicated an acceptable fit [29].

#### 2.5.4. Reliability Analysis

Internal consistency refers to how items designed to measure the exact construct yield consistent results [17]. In this study, Cronbach’s alpha was utilized to assess internal consistency. A coefficient value of ≥ 0.7 was considered satisfactory [17]. Also, the Spearman–Brown coefficient was used to test split‐half reliability [17]. A value from 0.5 to 0.7 was considered moderate, between 0.7 and 0.9 was considered good, and above 0.9 was considered excellent [30]. Furthermore, the intraclass correlation coefficient (ICC) was used to assess the reproducibility of the scale. Reproducibility was categorized into four levels based on ICC: “excellent” (ICC > 0.75), “good” (ICC ranging from 0.60 to 0.74), “fair” (ICC ranging from 0.40 to 0.59), or “poor” (ICC < 0.40) [31].

### 2.6. Sample Size

Based on the principle that the sample size for factor analysis is 5–10 times the number of items [32], given that the original HAQ has 15 items, the baseline sample size calculated in this study is 10 times the number of items (10 × 15 = 150). Considering the 15% invalid questionnaire rate [33], the adjusted minimum sample size was 173 (150 + 150 × 15% ≈ 173). Since this study simultaneously conducted EFA and CFA to ensure that both analysis methods could obtain robust results, the minimum sample size was further doubled to 346 people (173 × 2 = 346). However, given that this study was in the initial stage of a longitudinal study, all available baseline data (*N* = 2024) were included in the analysis. This large sample size greatly enhances the statistical power and generalizability of the research results [34].

### 2.7. Ethical Considerations

Ethical approval for this study was sought and obtained from the Human Ethics Review Committee at the Hong Kong Polytechnic University (reference number: HSEARS20241014001). The research aim was clearly explained to the participants, who then provided their written informed consent to participate. Participation was voluntary, and participants were informed of their right to withdraw from the project without penalty. The participants’ anonymity was maintained, and all their data remained confidential and accessible only to the researchers involved in this study.

## 3. Results

### 3.1. Descriptive Analysis

The sample consisted of 2024 valid responses in this cross‐sectional study. The participants’ ages ranged from 18 to 94 years old. Approximately one‐quarter (24.8%) of them were male, while the majority (73.3%) were female, and a small proportion (1.6%) declined to disclose their gender. Most participants (87.1%) did not live alone, with 12.7% living alone. In terms of educational level, the majority (42.9%) held bachelor’s degrees, while others had educational attainment ranging from secondary school or below (20.8%), to associate degrees (17.9%) and master’s degrees or above (18.1%). Regarding marital status, the majority were single (66.9%), while others were married (25.7%), divorced (2.5%), widowed (2%), or unwilling to disclose their marital status (2.7%). The majority of participants (64.8%) reported no medical conditions, while 21.6% had one medical condition, 8.2% had two medical conditions, and 5% had three or more medical conditions. The background characteristics of the participants are presented in Table 1.

**TABLE 1:** Characteristics of participants.

Characteristics	*n* (%)
Age, median (SD, quartiles (25%–75%), year	30 (17.2, 23–47)
Gender	
Male	504 (24.8)
Female	1484 (73.3)
Unwilling to disclose	33 (1.6)
Living alone	
Yes	257 (12.7)
No	1767 (87.1)
Educational level	
Secondary school or below	423 (20.8)
Associate degree	363 (17.9)
Bachelor’s degree	871 (42.9)
Master’s degree or above	367 (18.1)
Marital status	
Single	1357 (66.9)
Married	521 (25.7)
Divorced	51 (2.5)
Windowed	41 (2)
Unwilling to disclose	54 (2.7)
Medical condition	
0	1314 (64.8)
1	439 (21.6)
2	166 (8.2)
3 or more	105 (5)

### 3.2. Content Validity

The I‐CVI values ranged from 0.8 to 1.0, with an S‐CVI of 0.9, suggesting that the Chinese version of the HAQ demonstrated adequate content validity.

### 3.3. Item Analysis

The item analysis results for HAQ are presented in Table 2 from the initial stage. Q1 had a corrected item‐total correlation of less than 0.2. Q8 loading at Factors 1 and 3 was 0.360 and 0.354, respectively, while Q9 loading at Factors 1 and 4 was 0.529 and −0.305, respectively. After making final decisions on elimination, incorporating professional judgment and assessment, we eliminated Q1, Q8, and Q9. Factor analysis supported three components: (1) Physical/Mental and Sense of Purpose; (2) Engagement in Functional Activities; (3) Engagement in Social and Physical Activities.

**TABLE 2:** Fifteen‐item healthy aging questionnaire.

		Factor				Corrected item‐total correlation	Cronbach’s alpha (if item deleted)
		1	2	3	4		
Q1	Do you smoke?				0.906	0.039	0.824

Q2	Do you engage in at least one social activity each week?			0.753		0.350	0.813

Q3	Do you engage in at least one activity each week that you find meaningful?			0.690		0.324	0.815

Q4	In the past week, how often did you engage in exercise that made you sweat?			0.615		0.263	0.818

Q5	Do you find any of the following situations difficult due to physical health limitations?		0.711			0.300	0.815
	• Walk a few blocks (about 100–200 m) or						
	• Go home with groceries from the supermarket						

Q6	How often do you have a physical health issue that hinders you from working or performing other activities?		0.809			0.454	0.806

Q7	How often do you have physical health or emotional problems that hinder you from participating in social activities?	0.328	0.701			0.574	0.796

Q8	How many relatives or friends do you feel at ease with, are able to talk to about private matters, or can seek help from?	0.360		0.354		0.359	0.822

Q9	How would you rate the overall quality of the healthcare you receive?	0.529			−0.305	0.354	0.812

Q10	In the past year, did you consider yourself a happy person?	0.786				0.662	0.790

Q11	Do you forget your appointments?		0.585			0.301	0.816

Q12	“As I age, I feel more and more useless.”	0.545				0.508	0.807

Q13	Are you satisfied with your thinking ability?	0.669				0.537	0.800

Q14	How do you think your current mental health status compares to others your age?	0.859				0.718	0.782

Q15	Overall, how do you feel about your physical and mental health within your current age group?	0.841				0.732	0.781

### 3.4. Construct Validity and Model Fit

#### 3.4.1. EFA: Sample A

**FIGURE 1:**
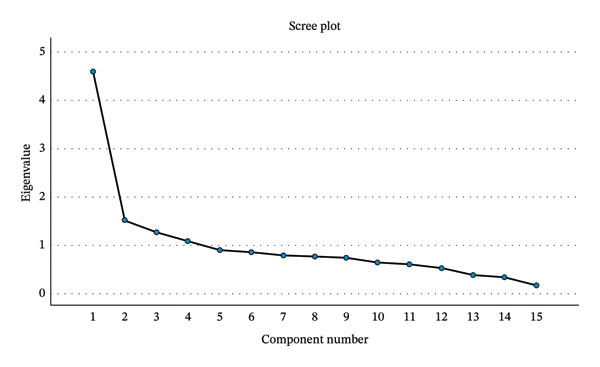
Scree plot of the 15‐item HAQ.

The underlying factor structure of the 15‐item scale was investigated using PAF. Data was analyzed from sample A (*n* = 1012). An initial analysis of the factor matrix revealed that all items loaded more than 0.3 in a four‐factor solution. The 15‐item scale was satisfactory using the PAF extraction procedure with a KMO measure of sampling adequacy of 0.852 and Bartlett’s test of sphericity statistically significant (Table 3). The scree plot indicated the clearest break after four factors (Figure 1). This four‐factor solution accounted for a total cumulative of 56.237%.

**TABLE 3:** Exploratory factor analysis (Varimax and Promax rotations) and internal consistency for the scale.

	Factor (Varimax rotation)			Factor (Promax rotation)			Corrected item‐total correlation	Cronbach’s alpha (if item deleted)
	1	2	3	1	2	3		
Q2			0.761			0.780	0.344	0.824
Q3			0.688			0.708	0.315	0.826
Q4			0.644			0.658	0.267	0.830
Q5		0.751			0.703		0.306	0.825
Q6		0.812			0.835		0.476	0.814
Q7		0.704			0.776		0.589	0.804
Q10	0.789			0.813			0.635	0.800
Q11		0.580			0.595		0.317	0.827
Q12	0.585			0.628			0.510	0.816
Q13	0.698			0.720			0.548	0.808
Q14	0.887			0.896			0.720	0.789
Q15	0.848			0.880			0.735	0.787

**FIGURE 2:**
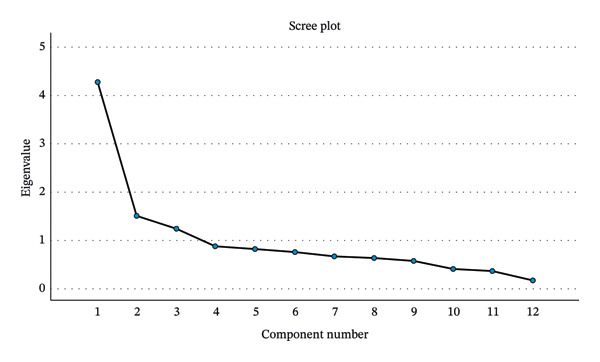
Scree plot of the 12‐item HAQ.

After removing Q1, Q8, and Q9, all items load more than 0.4 in a three‐factor solution. The dimension of the 12‐item scale was satisfactory using the PAF extraction procedure with a KMO measure of sampling adequacy of 0.841 and Bartlett’s test of sphericity statistically significant. The scree plot indicated the clearest break after three factors (Figure 2). This three‐factor solution accounted for a total cumulative of 58.178%. EFA with Promax rotation revealed a three‐factor solution, explaining 58.178% of the total variance. The inter‐factor correlations ranged from 0.219 to 0.406, indicating moderate associations among the domains. The three factors were interpreted as physical/functional health, psychological/social well‐being, and lifestyle/cognitive engagement (Table 4).

**TABLE 4:** Definitions and scoring of the Chinese HAQ‐12 subscales.

Subscale (factor)	Definition	Included items	Score range
Factor 1: Physical/Mental & Sense of Purpose	Reflects subjective appraisal of overall health, mental well‐being, and the psychological sense of meaning in life.	Q10, Q13, Q14, Q15	0–12
Factor 2: Engagement in Functional Activities	Measures the individual’s ability to perform activities of daily living and the extent to which physical health limits roles.	Q5, Q6, Q7, Q12	0–12
Factor 3: Engagement in Social & Physical Activities	Assesses active lifestyle choices, including physical exercise and participation in social/productive networks.	Q2, Q3, Q4, Q11	0–12
Total score	A comprehensive index of healthy aging status across multiple dimensions.	All 12 items	0–36

#### 3.4.2. CFA: Sample B

**FIGURE 3:**
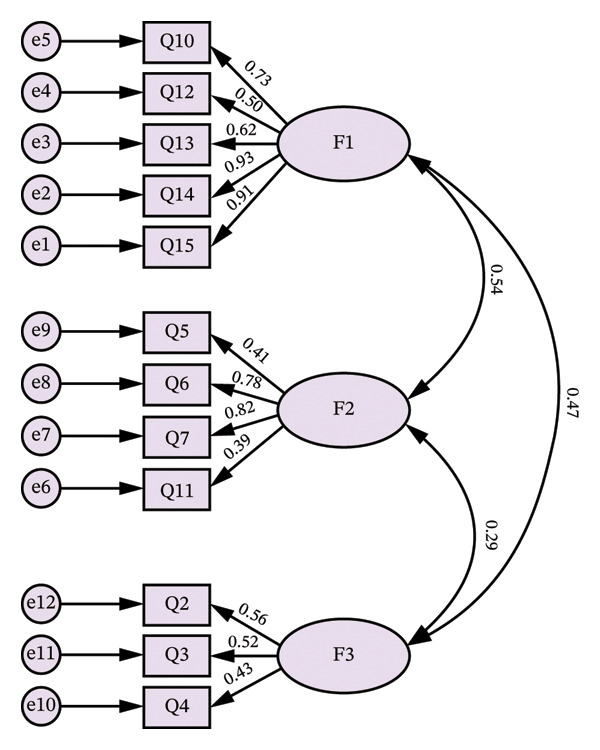
Confirmatory factor analysis of the three‐factor model of the Chinese version of the HAQ (*n* = 1012, group B).

A total of 1012 participants were included in the CFA. The 12‐item three‐factor model identified through the EFA in the previous process was tested using CFA (Figure 3).

RMSEA for the overall instrument was 0.060, indicating an acceptable fit [29]. Additionally, IFI was 0.956, TLI was 0.943, and CFI was 0.956, all of which suggest a good model fit (> 0.9) [27].

### 3.5. Reliability

Cronbach’s alpha for the 12‐item scale was 0.827, reflecting good internal consistency [18]. All 12 items had a corrected item‐total correlation of > 0.2. Apart from Q4, none of the alpha values increased when the item was deleted. Although the change in alpha after deleting Q4 was minimal, the research team decided to retain this item following discussions with experts. The split‐half reliability of the Chinese HAQ was assessed using the Spearman–Brown coefficient, which yielded a value of 0.697, indicating moderate reliability. Additionally, the ICC value of 0.630 indicates that the HAQ shows good reproducibility, making it suitable for further research and practical applications.

A comprehensive summary of the psychometric evaluation for the Chinese HAQ (*N* = 2024), including reliability and validity, is presented in Table 5.

**TABLE 5:** Summary of psychometric properties of the Chinese version of HAQ (*N* = 2024).

Property	Metric/method	Results	Interpretation
Content validity	I‐CVI (Item‐level) <br> S‐CVI (scale‐level)	I‐CVI: 0.80 – 1.0 <br> S‐CVI: 0.90	Adequate: The items were deemed relevant and appropriate by a panel of 7 experts.
Construct validity (EFA)	Principal axis factoring (Sample A, *n* = 1012)	3‐factor structure; 58.18% cumulative variance explained	Strong: Items loaded clearly into three dimensions after removing Q1, Q8, and Q9.
Construct validity (CFA)	Fit indices (Sample B, *n* = 1012)	RMSEA: CFI = 0.956, IFI = 0.956, RMSEA = 0.060	Good to Excellent: All indices met the criteria for a good model fit (CFI/IFI > 0.95; RMSEA < 0.08).
Internal consistency	Cronbach’s alpha	0.827	Good: Indicates high internal consistency and that items measure the same construct.
Split‐half reliability	Spearman–Brown coefficient	0.697	Moderate: Suggests acceptable consistency between the two halves of the instrument.
Reproducibility	Intraclass correlation coefficient (ICC)	0.630	Good: Indicates the instrument yields stable and reproducible results.
Responsiveness	Longitudinal sensitivity to change	Not assessed	N/A: The study was cross‐sectional. The authors recommend longitudinal studies to assess this.
Evidence quality	Methodological rigor	Large sample size (*N* = 2024); followed STROBE guidelines	High internal validity: Use of separate samples for EFA and CFA prevents overfitting and increases generalizability.

## 4. Discussion

Healthy aging has become an important priority, particularly as global populations age rapidly. It is recognized that promoting healthy aging improves the quality of life for older adults, helps reduce healthcare costs, and fosters greater societal participation [1]. The HAQ, originally developed and validated in Singapore, has proven effective in assessing various dimensions of healthy aging, such as physical function, cognitive health, emotional well‐being, and social engagement, particularly among individuals aged 55 and above [6]. However, there remains a gap in the literature regarding the applicability of this tool to a broader adult population, particularly those aged 18 and older. The present study validated the HAQ within the Hong Kong context to bridge this gap, focusing on adults across a wider age range. This extension of the HAQ’s use to a younger adult demographic allows for a more comprehensive understanding of the healthy aging process throughout the adult life course, rather than solely focusing on older adults.

According to EFA, double loadings were expected and tolerated, as they suggested a conceptual overlap between factors and items. However, such overlap complicated the distinction between factors and negatively affected the model’s fit [35]. Despite the items in the Chinese version of the HAQ being considered relevant and culturally appropriate during the content validity assessment, the EFA revealed that Q8 and Q9 did not meet the criteria for inclusion in the final questionnaire version. Consequently, these items were removed due to their poor performance in the factor analysis. Q8 asked how many relatives or friends you feel at ease with or could talk to about private matters and/or call for help. This item proved challenging in terms of cultural interpretation and relevance. The concept of social support and intimacy can vary across age groups and cultural contexts, leading to differing interpretations of what constitutes close support [36]. Q8 loaded onto two factors: Physical/Mental and Sense of Purpose and Engagement in Social and Physical Activities. This cross‐loading suggests that the item may measure overlapping constructs rather than a distinct dimension. The dual association indicates that social support may be conceptually linked to psychological well‐being [37] and social engagement [38], making it difficult to categorize the item under a single factor. This ambiguity in factor structure contributed to the decision to remove Q8, ensuring greater conceptual clarity in the final version of the questionnaire.

Question 9 asked respondents to rate the overall quality of healthcare they received. While healthcare quality was an important aspect of healthy aging in older adults [39], this item did not perform well in the EFA of this study. Several reasons can explain this phenomenon. First, various personal factors, such as health status, access to medical resources, and attitudes toward medical services [40], may influence participants’ responses to this item. These factors may lead to significant differences in respondents’ perceptions of medical quality, making it difficult to use this item to consistently measure a single construct related to healthy aging. Secondly, while medical quality is important in healthy aging, especially for older adults, people’s perceptions of it may vary significantly across age groups, socioeconomic status, and healthcare systems [41].

In this study, the sample covered a wider age range (adults aged 18 years and above), and younger adults may have different perceptions of medical quality than older people. For younger adults, medical quality may not be a key issue in their daily lives because they may not face the same health challenges or frequent medical needs as older people [42]. Therefore, their perceptions of medical quality may be influenced by factors such as overall satisfaction with existing medical services rather than experiences related to age‐related health problems. In contrast, older adults, especially those with chronic diseases, may experience healthcare more directly and frequently, which makes the concept of healthcare quality more meaningful and closer to their lives. Q9 suffered from conceptual ambiguity because it asked respondents to rate “overall quality,” which different individuals could understand differently. Participants in this study answered this question based on their most recent healthcare experience, while others may think about their overall attitude toward the healthcare system. This conceptual ambiguity may lead to cross‐loadings and unclear relationships between this item and other factors in the model. As a result, Q9 was removed to maintain the focus of the questionnaire on the core dimensions of healthy aging.

The CFA results provided valuable insights into the structural validity of the Cantonese version of the HAQ. The model tested in the CFA was based on the three‐factor structure identified during EFA, which was composed of physical health, mental well‐being, and social engagement. The fit indices for the CFA showed a good model fit, indicating that the model fitted the data well according to established criteria [27]. The three‐factor solution identified in the CFA is consistent with the underlying structure suggested by the EFA. It also demonstrated that the 12‐item HAQ provides a reliable and valid measure of healthy aging. These results confirmed the robustness of the factor structure and its appropriateness for measuring the intended dimensions of healthy aging in the Hong Kong context.

The reliability analysis of the Chinese version of the HAQ showed promising results. Cronbach’s alpha was 0.827, indicating strong internal consistency. This meant the items reliably measured the same concept of healthy aging. This result was comparable to the Cronbach’s alpha of 0.735 found in the original Singapore version, which also demonstrated good internal consistency of the scale [6]. The split‐half reliability was 0.697, showing moderate reliability. This suggests that the tool performs consistently across different samples. Additionally, the ICC was 0.630, indicating good reproducibility. This means the questionnaire yields stable results over time. These findings confirm that the Chinese HAQ is a reliable tool for assessing healthy aging.

While the final 12‐item HAQ validated in this study prioritizes psychometric validity, we recognize that certain environmental indicators, such as social support and healthcare quality, are important for a comprehensive understanding of healthy aging. Recent cluster‐based research using the full 15‐item HAQ in Hong Kong [43] demonstrated that these items remain highly discriminative in profiling healthy aging subgroups, supporting their ongoing relevance. Although our study benefits from a large sample size and robust psychometric analysis, the sample was predominantly younger, female, and highly educated. This demographic skew may limit the generalizability of our findings to older adults and other groups. However, recent cluster‐based research using the same HAQ and cohort demonstrated that the instrument is capable of distinguishing healthy aging profiles across age, gender, and education levels [43]. This study has several limitations that should be considered when interpreting the findings. First, the sample predominantly consisted of young adults, with a median age of 30 years, a higher proportion of females, and a more educated population, which may not fully capture the experiences of older adults, who may have different perceptions of healthy aging. However, recent cluster‐based research using the same HAQ and cohort demonstrated that the instrument was capable of distinguishing healthy aging profiles across the adult life course [43]. Although efforts were made to ensure a diverse sample across age groups, further research with a larger proportion of older adults would help to better understand the applicability of the Chinese version of the HAQ in this group. Second, while the Chinese version of the HAQ demonstrated good content validity and internal consistency, the construct validity could be further explored. The EFA and CFA revealed a three‐factor structure, but it is important to note that the conceptual overlap observed in some items, such as Q8 and Q9, may impact the precision of measuring distinct dimensions of healthy aging. The decision to remove these items was based on their poor performance in factor analysis, but further testing with different samples or populations would be helpful to confirm the instrument’s construction validity across various contexts. Third, although Cronbach’s alpha and split‐half reliability were satisfactory, the moderate reliability of split‐half reliability (0.697) and the ICC of 0.630 suggests room for improvement. The moderate split‐half reliability indicated that the tool performed inconsistently when divided into two halves. Therefore, future studies could explore additional measurements to improve the instrument’s stability across different groups and time points. Also, while the overall Cronbach’s alpha of 0.827 indicates good internal consistency, further validation in diverse population groups was required to ensure the instrument holds up in different settings. Fourth, a cross‐sectional design limits the ability to conclude causality or the long‐term stability of the constructions assessed by the HAQ. Longitudinal studies would be beneficial in assessing how the factors measured by the HAQ change over time and how these changes relate to the health outcomes in different stages of life. Fifth, and importantly, this cross‐sectional validation study does not establish criterion validity. We did not correlate the Chinese HAQ scores with established clinical measures (e.g., frailty scales, activities of daily living assessments, or cognitive batteries) to demonstrate concurrent validity. Furthermore, the design does not allow for the assessment of predictive validity of the HAQ to forecast future aging‐related outcomes such as functional decline, hospitalization, or mortality. This represents a key direction for the next phase of research. Finally, although gender‐based analyses using the same dataset have shown comparable psychometric properties of the HAQ across male and female subgroups in separate research, formal measurement invariance testing across age and sex was not conducted in this study. Future research should employ multigroup confirmatory factor analysis to rigorously establish whether the instrument functions equivalently across demographic subgroups.

## 5. Implications

This study successfully translated and validated the Chinese version of the HAQ for use in Hong Kong’s adult population. The findings demonstrated that the HAQ possesses good content validity, internal consistency, and construct validity, confirming its reliability as a tool for assessing healthy aging. While the study provided strong evidence for the HAQ’s psychometric soundness, limitations such as the predominantly younger sample and the cross‐sectional design highlight the need for further research. Future studies should focus on validating the HAQ in young populations, improving its reliability, and assessing its applicability in longitudinal research. In addition, consideration should be given to adding more items or modules to the HAQ to incorporate environmental and contextual factors, ensuring full alignment with the WHO framework for healthy aging. Overall, the validated Chinese HAQ was a valuable tool for researchers, healthcare professionals, and policymakers to better understand and promote healthy aging across different stages of adulthood in Hong Kong.

A comprehensive flow diagram of the experimental procedure is provided in the Supporting Information (available here).

## Funding

No funding was received for this manuscript.

## Conflicts of Interest

The authors declare no conflicts of interest.

## Supporting Information

Participant Flow Diagram.

## Supporting information


**Supporting Information** Additional supporting information can be found online in the Supporting Information section.

## Data Availability

The data that support the findings of this study are available upon request from the corresponding author. The data are not publicly available due to privacy or ethical restrictions.
