# Supplementary material for: Validation and Evaluation of the Chinese Version of the Healthy Aging Questionnaire in Hong Kong
Source: J Aging Res. 2026 Apr 8;2026:3632426. doi: 10.1155/jare/3632426 (PMC13058564; doi:10.1155/jare/3632426)
Supplement: Supplementary file 1 — Supporting Information Additional supporting information can be found online in the Supporting Information section. [file JARE-2026-3632426-s001.docx]

**Participant Flow Diagram**

**Exclude**

- Internal testing responses: 4
- Duplicate entries: 90
- Withdrew consent: 1
- Missing data (>0.05%): 1

**Final Analytic Sample (**$\boldsymbol{N=2,024}$**):**

- - - Used for Psychometric Validation.
    - Sample A (EFA): $n=1,012$
    - Sample B (CFA): $n=1,012$

**Screening**

Total responses collected ($N=2,120$) (Online responses: 2,050 and Paper surveys: 70

**Identification**

online (social media, email) and offline (community centers, posters)
